# Cholesterol Induces Uneven Curvature of Asymmetric Lipid Bilayers

**DOI:** 10.1155/2013/965230

**Published:** 2013-05-16

**Authors:** S. O. Yesylevskyy, A. P. Demchenko, S. Kraszewski, C. Ramseyer

**Affiliations:** ^1^Department of Physics of Biological Systems, Institute of Physics of the National Academy of Sciences of Ukraine, Prospect Nauky 46, Kiev 03680, Ukraine; ^2^A.V. Palladin Institute of Biochemistry, National Academy of Sciences of Ukraine, Leontovicha street 9, Kiev 01601, Ukraine; ^3^Nanomedicine Laboratory, Imagery & Therapeutics, EA4662, Université de Franche-Comté, Centre Hospitalier Universitaire de Besançon, 16 Route de Gray, 25030 Besançon, France

## Abstract

A remarkable flexibility is observed in biological membranes, which allows them to form the structures of different curvatures. We addressed the question of intrinsic ability of phospholipid membranes to form highly curved structures and the role of cholesterol in this process. The distribution of cholesterol in the highly curved asymmetric DOPC/DOPS lipid bilayer was investigated by the coarse-grained molecular dynamics simulations in the membrane patches with large aspect ratio. It is shown that cholesterol induces uneven membrane curvature promoting the formation of extended flattened regions of the membrane interleaved by sharp bends. It is shown that the affinity of cholesterol to anionic DOPS or neutral DOPC lipids is curvature dependent. The cholesterol prefers DOPS to DOPC in either planar or highly curved parts of the membrane. In contrast, in the narrow interval of moderate membrane curvatures this preference is inverted. Our data suggest that there is a complex self-consistent interplay between the membrane curvature and cholesterol distribution in the asymmetric lipid bilayers. The suggested new function of cholesterol may have a biological relevance.

## 1. Introduction

The curvature is an important characteristic of the biological membranes. Different parts of the cell membrane form invaginations and protrusions ranging from small random undulations to large and highly organized structures [1]. The origins of the membrane curvature are diverse. First of all, each type of the membrane lipids possesses the so-called spontaneous curvature, which originates in the difference of relative volumes of the lipid headgroups and tails [2]. The overall shape of the lipid can be described schematically as a prism. Packing of such prisms produces either concave or convex monolayer [3, 4]. Since the lipid compositions of the membrane leaflets are different, the whole bilayer may develop a curvature. Other sources of curvature are peripheral and integral membrane proteins and the elements of cytoskeleton [5]. It is established that the membrane curvature plays an important role in the membrane fusion [4, 6], endocytosis [7], mechanosensitivity of the sensory cells [8] and separation of various membrane components [9] and thus is of great practical importance.

In addition to the curvature, the eukaryotic cell membranes are highly asymmetric. It is well known that plasma membranes of the phosphatidylcholine (PC) and sphingomyelin (SM) are located predominantly in the outer leaflet, whereas most of phosphatidylethanolamine (PE) and practically all phosphatidylserine (PS) and phosphoinositides are located in the inner leaflet [14, 15]. Phospholipid asymmetry is maintained due to the action of specific lipid-translocating ATPases [16]. Spontaneous transmembrane diffusion (flip-flop) of phospholipids is extremely slow due to high barrier of translocating their highly polar heads through the membrane interior.

The picture becomes even more complex if cholesterol distribution in the membranes is taken into account. Compared to phospholipids, cholesterol has much lower free energy barrier of flip-flop transitions between the membrane leaflets and higher lateral mobility [16]. The distribution of cholesterol in asymmetric and curved membrane cannot be even [1, 2, 17]. Cholesterol has stronger affinity to phospholipids with saturated chains [3]. Length and saturation of the lipid tails and the size of their heads may also influence its distribution [2, 4]. The hydrogen bonding with SM located at the outer leaflet can explain stabilization by cholesterol of lipid microdomains (rafts) and suggests its partial (or possibly predominate) location at this leaflet [5]. On the other hand, small head size of PE and repulsive interactions between PS charged groups can favor easier hydrogen bonding with deeper penetrated water molecules and thus enhance location of cholesterol at the inner leaflet. 

In general existing experimental techniques cannot determine cholesterol distribution in the asymmetric membranes with great fidelity [6, 7]. Despite these limitations an important role of cholesterol in controlling the membrane curvature and asymmetry is well recognized. Among the most general conclusions is the dependence of the membrane-bending rigidity on cholesterol concentration. The membranes with higher cholesterol content require more energy to bend [8].

**Figure 1:**
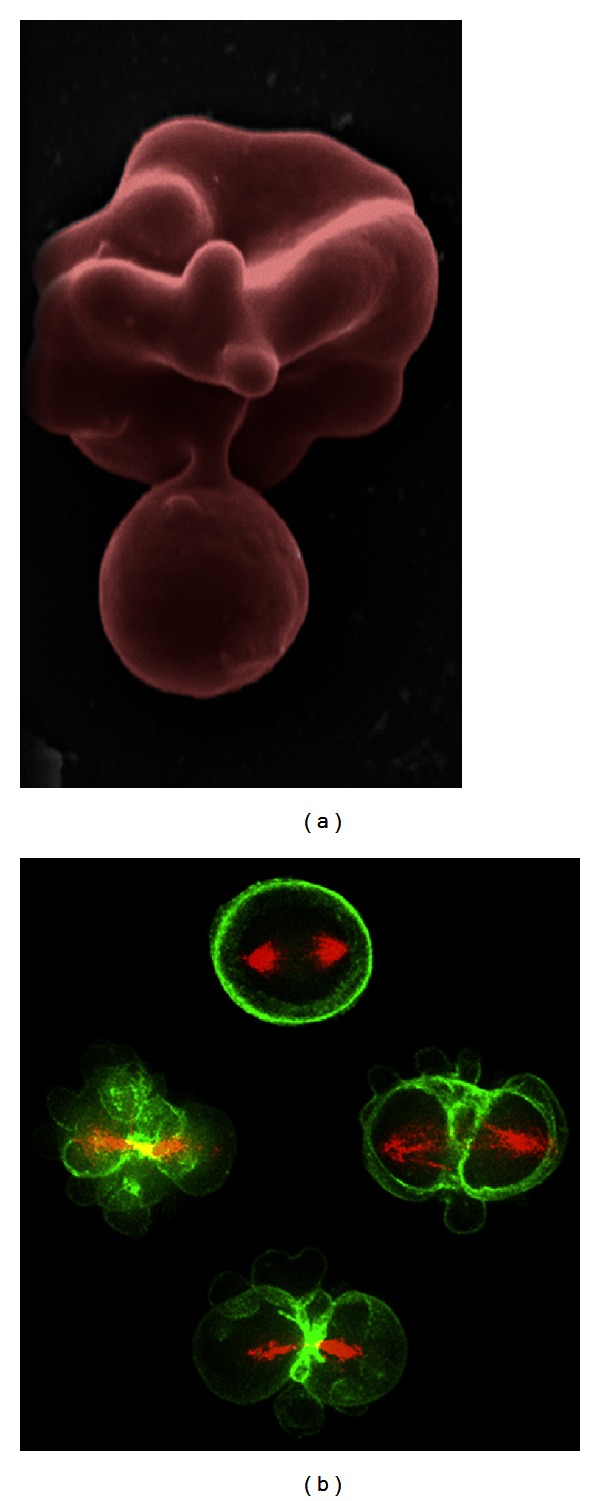
Examples of highly curved membrane blebs formation in living cells during reticulocyte enucleation [10] (a) and during the mitosis of *Drosophila* S2 cell [11] (b).

There are many experimentally observed cellular phenomena, which are likely to be governed or influenced by phospholipid asymmetry, membrane curvature, and cholesterol distribution [9]. Such phenomena include the formation of synaptic vesicles [18], formation of apoptotic bodies [19, 20], the processes of membrane fusion [21, 22], and budding of enveloped viruses from the plasma membrane [23, 24]. Another intriguing phenomenon is the formation of blebs—small spontaneous bulges on the membrane, which lack support of filaments or cytoskeleton proteins and often observed during apoptosis [19, 20] blood cell maturing [10] and mitosis [11] (Figure 1). The formation of the blebs is likely to be governed by the intrinsic factors such as membrane curvature, asymmetry of lipid composition, and cholesterol content, which makes them a convenient playground for studying the interplay of these factors.

Cholesterol affects the conformation of membrane fusion proteins, such as the HIV-1 gp41 fusion domain [25, 26], which are known to induce the membrane curvature. It was shown recently that cholesterol also regulates membrane penetration depth and occupied surface area of the gp41 fusion domain and thus can control curvature in real biological membranes during membrane fusion events [27, 28].

It is established that the membrane proteins play an important role in determining lateral cholesterol distribution in the membranes. Certain types of proteins and lipoproteins are knows to sequester with cholesterol and to stabilize cholesterol-rich domains [29]. Such proteins usually contain cationic clusters, myristoylation, or CRAC motif, which are recognized as cholesterol-sequestering factors [29].

Studies of cholesterol-rich membrane microdomains (rafts) are of great interest for biomedical applications. Particularly, the respiratory syncytial virus (RSV), which causes respiratory infections in children, binds to cholesterol-rich lipid rafts in the plasma membrane of human bronchial epithelial cells [30]. Moreover, the release of RSV virus particles from infected cells also requires cholesterol-rich lipid rafts [31]. Depletion of cholesterol concentration profoundly inhibited RSV infection in the cell cultures [30]. It was also shown that cholesterol deficiency disease in mice leads to malformation of secretory granules and to decrease of the membrane curvature in pancreatic cells [32].

The facts that the results of experiments on cholesterol partitioning and dynamics in lipid bilayers are so challenging and contradicting stimulated numerous computational studies. The most popular method of simulating biological membranes is molecular dynamics (MD) [33, 34]. Classical Newton's equations of motion are solved iteratively in MD providing the trajectories of all atoms in time. Applying the concepts from statistical mechanics, the resulting trajectories can be used to evaluate various static and time-dependent structural, dynamic, and thermodynamic properties of the system. MD simulations provide a unique opportunity to study various aspects of the membrane functioning at the all-atom level of details, which is not accessible and will probably never be accessible for experimental techniques [33, 34]. 

Recently all-atom molecular dynamics (MD) simulations were used to study cholesterol interactions with the lipids [35], the condensing effect of cholesterol [36–38], and the properties of cholesterol-induced rafts [39]. There were attempts of obtaining the potentials of mean force (PMFs) for transferring cholesterol from lipid bilayers to water [40]. Despite significant insights obtained in these studies, the time scale of cholesterol redistribution between the monolayers was beyond the reach for all-atom simulations. 

The coarse-grained (CG) models are widely used in MD studies of the membranes in order to increase the time and length scale of simulations. In these models the groups of adjacent atoms are combined into the “beads,” which interact with each other by means of empirical potentials. Since the number of the beads is much smaller than the number of individual atoms, significant speedup of computations could be achieved. The coarse graining provides the nanoscopic description of the studied systems instead of purely atomistic treatment [41].

**Figure 2:**
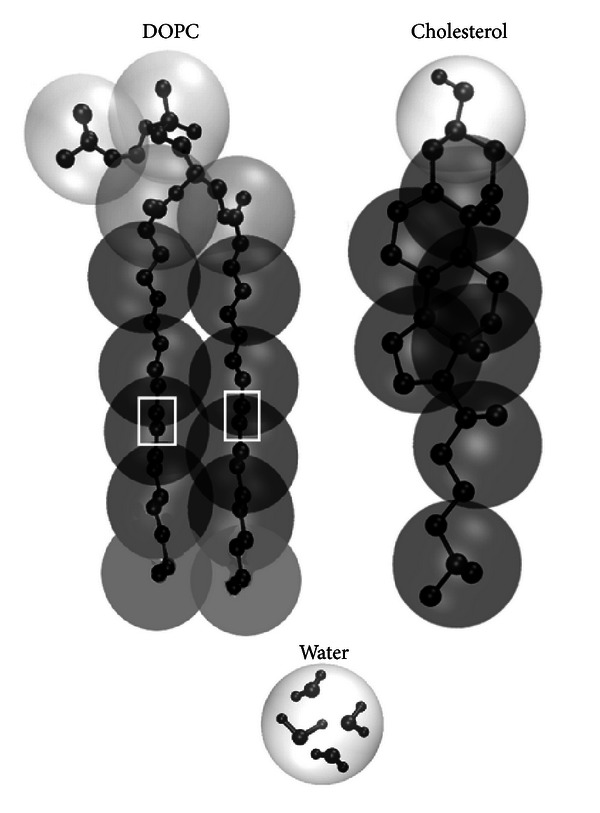
The scheme of coarse graining used in MARTINI model [12, 13]. Coarse-grained particles are shown as semitransparent spheres and superimposed into the atomistic representations of the corresponding molecules. White rectangles indicate double bonds in the lipid tails. The figure is adapted from MARTINI web site http://md.chem.rug.nl/cgmartini/index.php/about/martini.

One of the most popular CG models for membrane simulations is MARTINI force field [12, 42–44]. On average, in MARTINI one CG particle represents four heavy atoms with associated hydrogens (Figure 2). MARTINI model was recently used in extensive study of cholesterol behavior in symmetric lipid membranes [13] and in the branched bilayer systems [45]. The effects of the cholesterol-dependent phase separation and lipid sorting were found. Particularly, it was shown that cholesterol migrates to either curved or planar regions of the membrane depending on the lipid composition [45].

Despite the notable progress of MD simulations of lipid bilayers, surprisingly small attention is paid to membrane asymmetry and curvature associated with this asymmetry. The distribution of cholesterol in relation to its functional role in such membranes still did not attract the necessary attention, especially in the computational studies.

Modern MD simulations of the lipid membranes are usually performed on the relatively small patches of planar lipid bilayers. Existing attempts of simulating highly curved membranes originate mostly from the membrane fusion studies [4, 18, 19] and the studies of mechanosensitive ion channels [20]. Significant curvature is also observed in the simulations of bilayers with asymmetric lipid composition [21]. Although there are few MD studies of the curved membranes now, there is no doubt that such systems would become increasingly popular in the coming years due to practical importance of the membrane curvature effects.

In this work we address the problem of mutual influence of the membrane curvature and cholesterol distribution on highly curved asymmetric lipid membrane using coarse-grained MD simulations. We show that spontaneous membrane curvature is significantly modulated by cholesterol. Particularly it is shown that cholesterol induces uneven curvature and accumulates in both planar and highly curved regions of the membrane. Methodological issues in MD simulations of highly curved asymmetric membranes are discussed as well as special procedures of data analysis.

## 2. Methods

### 2.1. Simulation Setup

 Lipid bilayers were modeled using the coarse-grained MARTINI force field version 2.1 [12]. All simulations were performed with the GROMACS 4.5.5 software package [46] at the temperature of 320 K. Recommended simulation parameters for MARTINI force field were used [12]. In all simulations a Berendsen barostat with the relaxation constant of 5 ps was used for semi-isotropic pressure coupling. The pressure of 1 atm was maintained separately in *XY* plane and in *Z* direction. 

**Figure 3:**
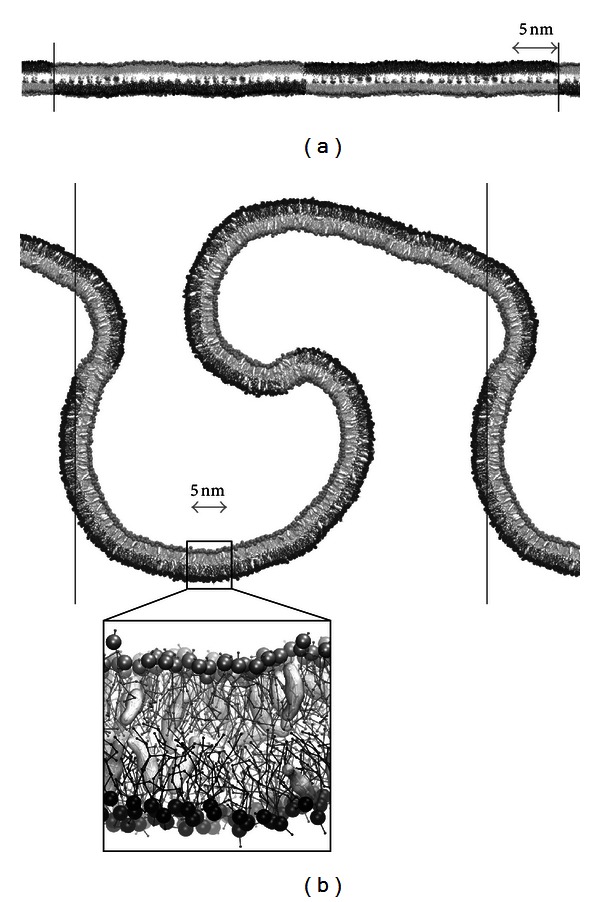
The shape of the model membrane with cholesterol. (a) Initial planar bilayer. (b) Final curved bilayer after 20 *μ*s of simulation. The scales of the panels (a) and (b) are shown. DOPC lipids are black, DOPS lipids are gray, and cholesterol molecules are white and are shown in surface representation. Phosphate groups of the lipids are shown as large spheres. Vertical lines show the extents of the periodic simulation box in *X* dimension. The system without cholesterol looks very similar.

An asymmetric membrane containing neutral dioleoylphosphatidylcholine (DOPC) or anionic dioleoylphosphatidylserine (DOPS) lipids was constructed. Each monolayer of the model membrane consists of two rectangular blocks of lipids of different type. Positions of the blocks in the second monolayer are inverted with respect to the first one. As a result, the whole system consists of two inverted asymmetric bilayer patches stacked side by side (Figure 3(a)). The lateral dimensions of the blocks of different lipids changed in the course of simulations in order to optimize their areas per lipid. Thus no surface tension stress is developed in the monolayers. However, each monolayer becomes inhomogeneous and the membrane could develop significant curvature caused by different intrinsic curvatures of different lipid blocks (Figure 3(b)). The system contained 4032 lipids (2016 lipids of each type), ~600000 coarse grained water particles, and 2016 coarse grained sodium ions. The lipids were initially arranged into the planar elongated rectangular membrane patch with the dimensions 111.0 × 12.4 nm in *XY* plane. It is necessary to note that the properties of the membrane patch, which can bend in *X* dimension only, may not be identical to the properties of real membrane, which is free to deform in both *X* and *Y* dimensions. However, the width of the membrane patch is large enough to eliminate the direct influence of the periodic boundary conditions; thus, the differences should be minor.

Artificial repulsive potential was introduced between the coarse-grained phosphate beads of the lipids from different blocks to prevent lateral mixing of DOPC and DOPS lipids. Lenard-Jones potential with the parameters c6 = 0, c12 = 2.581 (GROMACS force field units) was used to implement this repulsion. 

The CG model of cholesterol designed for MARTINI force field [47] was used. Cholesterol molecules were placed in the central plane of bilayer, oriented in *XY* plane, and distributed evenly on rectangular grid with the account of periodic boundary conditions in *XY* plane. After that the monolayers were moved into opposite directions on *Z* scale for eliminating any steric overlaps of the lipid tails with cholesterol molecules. This forms a gap in the center of bilayer, which accommodates flat layer of aligned cholesterol molecules (Figure 3(a)). The number of cholesterol molecules was 784 (cholesterol to lipid ratio ~ 0.2). The same system without cholesterol was also simulated as a reference.

The system with cholesterol was simulated for effective time of 20 *μ*s, while the system without cholesterol simulated for effective time of 23 *μ*s. The effective time scale of CG simulations of lipids with MARTINI force field is proved to be 4 times longer than the actual simulation time [12]. The effective simulation time is used hereon. Only equilibrated parts of trajectories were used for analysis.

### 2.2. Analysis of the Curved Membranes

 The primary difficulty in the analysis of curved bilayers is the absence of global membrane normal, which could be used across the whole simulation box. The shape of the curved membrane should be first parameterized by some analytical surface. After that, normals to this surface can be determined and all subsequent analysis should be performed in terms of the local normal at the point of interest. Quantitative measure of the local curvature is also of interest and should be determined at each point of the membrane.

 In our knowledge no tools for such analysis exist in commonly used MD packages and molecular visualization programs, such as GROMACS [22], NAMD [23], AMBER [24], VMD [10], and PyMol [11]. All existing tools are based on the assumption that the membrane is planar and the membrane normal is oriented along *Z* axis of the simulation box, which is obviously not the case for the curved membrane. Particularly there are no tools for determining the shape of the membrane in the case of significantly curved bilayer. It is also not possible to determine the membrane curvature quantitatively at particular point or to compute mean membrane curvature.

 We assume that the membrane is significantly curved in the *XZ* plane and remains almost planar in *Y* direction. This assumption is always valid if *Y* dimension of the simulation box is small in comparison with *X* and *Z* dimensions, and the shape of the membrane can be described accurately by the two-dimensional curve instead of the three-dimensional surface. 

 The principal scheme of this algorithm is the following:

1. assigning the lipids to one of the monolayers,
2. determination of the membrane midline in *XZ* plane as a set of discrete points equally spaced along the membrane,
3. approximation of the membrane midline by analytical curve,
4. computation of the membrane curvature in each discrete point along the midline.

Step 1 is performed only once for the first frame of MD trajectory because no spontaneous flip-flop transitions of the lipids are usually observed in MD simulations. Steps 2–4 should be performed for each trajectory frame.

In order to perform an assignment of the lipids to monolayers a single marker atom is chosen in the head group of each lipid (the P atom of the phosphate group in our case). The graph of the nearest neighbors is constructed by connecting all marker atoms situated within the cut-off distance *r*_*p*_ from each other (the value of *r*_*p*_ = 2.0 nm is chosen empirically). After that, two disjoint sets of vertexes, which represent two monolayers, are found on this graph.

 In order to determine the membrane midline one needs to know how to compute the local center of bilayer and the local bilayer normal in any given point. We used the procedure developed in our previous work [7], which takes an arbitrary point $\vec{p}$ and returns local center and the normal of bilayer $\vec{c},\;\;\vec{n}=\text{Local}\left(\vec{p}\right)$. The reader is referred to [7] for the details.

Determination of the membrane midline is performed as follows.

1. The marker headgroup atom of arbitrary lipid is chosen.
2. Local membrane normal ${\vec{n}}_{1}$ and local bilayer center ${\vec{c}}_{1}$ are computed for this marker atom. ${\vec{c}}_{1}$ is now the first point of the membrane midline.
3. The approximate position of next *k* + 1 point of the midline is obtained as ${\vec{c}}_{k+1}^{\;\;\;\;\;\;\;\;\;\;\text{appr}}={\vec{c}}_{k}+\left({\vec{n}}_{k}\cdot \vec{j}/||{\vec{n}}_{k}\cdot \vec{j}||\right)s$, where $\vec{j}$ is the unit vector in *Y* direction (perpendicular to the working *XZ* plane), *s* is a discrete spacing between the midline points (the value of 1 nm is used in this work), dot stands for vector product, and double braces denote the vector norm. In other words, the next point is shifted from ${\vec{c}}_{k}$ by the distance *s* in tangential direction according to the current normal ${\vec{n}}_{k}$.
4. The point ${\vec{c}}_{k+1}^{\;\;\;\;\;\;\;\;\;\text{appr}}$ is somewhat shifted from the true membrane midline because it was computed using ${\vec{c}}_{k}$ and ${\vec{n}}_{k}$ at the previous point. True membrane normal ${\vec{n}}_{k+1}$ and local bilayer center ${\vec{c}}_{k+1}$ are computed as ${\vec{c}}_{k+1},\;\;{\vec{n}}_{k+1}=\text{Local}\left(\;\;\;\;\;\;{\vec{c}}_{k+1}^{\;\;\;\;\;\;\;\;\;\;\text{appr}}\right)$, and ${\vec{c}}_{k+1}$ is used as *k* + 1 midline point.
5. The distance *d* between the current ${\vec{c}}_{k+1}$ and initial ${\vec{c}}_{1}$ midline points is computed taking into account periodic boundary conditions in the simulation box (the distance between the closest periodic images of both points is computed). If *d* < *s* and *k* > 1, then the midline points covered the whole membrane, thus finish the computation. If not, increase *k* by one and go to step 3.

It is more convenient to represent the membrane midline as the separate parametric dependencies of *X* and *Z* coordinates *X*(*l*_*i*_) = *c*_*i*_^(*x*)^, *Z*(*l*_*i*_) = *c*_*i*_^(*z*)^ where *l*_*i*_ = *is* is the length of the membrane measured from the starting point with respect to the periodic boundary conditions of the simulation box, *i* = 1,2 …, *N*, where *N* is the number of midline points.

The midline is approximated by the sum of cosines by means of the fast Fourier transform (FFT) [25]. Performing FFT on *X*(*l*_*i*_) and *Z*(*l*_*i*_) gives corresponding discrete complex value spectra in the wavenumber domain *S*_*X*_(*λ*_*i*_) and *S*_*Z*_(*λ*_*i*_), where *λ*_*i*_ = *N*/*is* is the wavelengths of *N* discrete spectral components [25].

The curvature *κ* of the flat parametric curve is given by the standard expression [26]:

(1) $$\begin{array}{c} \kappa \left(l\right)=\frac{\dot{X}\left(l\right)\ddot{Z}\left(l\right)-\dot{Z}\left(l\right)\ddot{X}\left(l\right)}{{\left(\dot{X}{\left(l\right)}^{2}+\dot{Z}{\left(l\right)}^{2}\right)}^{3/2}}, \end{array}$$

where dots denote the derivatives with respect to *l*. In our case the derivatives could be computed by multiplying corresponding spectra by *j*/*λ* in the wavenumber domain and performing inverse FFT [25].

 In practical computations the spectra *S*_*X*_(*λ*_*i*_) and *S*_*Z*_(*λ*_*i*_) contain a number of the short wavelengths components, which produce large chaotic oscillations of derivatives and *κ*(*l*). Such artifacts could be eliminated by performing low-pass filtering of the spectra and keeping only the components with *λ* > *λ*_*c*_. The value of the cut-off wavelength *λ*_*c*_ should be chosen empirically for each particular membrane system. In this work the value *λ*_*c*_ = 15 nm was chosen.

**Figure 4:**
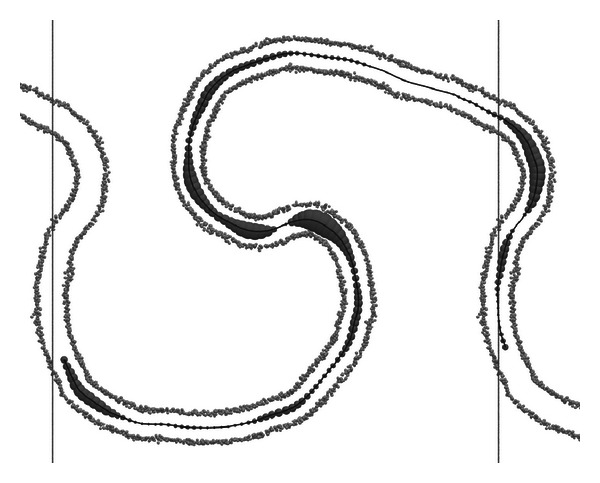
Visualization of the bilayer midline and the curvature at *t* = 20 *μ*s in the simulation with cholesterol. The phosphate coarse grained particles of both monolayers are shown as small spheres. The membrane midline is shown as a solid black line. The radii of the gray spheres drawn on the midline are proportional to the absolute value of the membrane curvature in the given point. Vertical lines show the size of the periodic simulation box in *X* dimension.

Figure 4 shows the membrane midline and the membrane curvature computed with our algorithm for one of trajectory frames. It is clearly seen that the membrane midline is reconstructed precisely and that the membrane bends are correctly recognized as the regions of large curvature.

The algorithm developed above was implemented in C++ programming language using Pteros molecular modeling library [27].

### 2.3. Measuring Flip-Flop Time

 Large simulation time allows observing multiple flip-flop transitions of cholesterol directly. Only the transitions, which occur between the domains consisting of different lipids, were considered. This allows disregarding those regions of the bilayer, in which the lipids of the same type appear in both monolayers due to random lateral diffusion of the lipid domains and as a result of adjusting their areas in the course of simulations. The time between two subsequent flip-flop events was recorded separately for each cholesterol molecule. The histogram of the intervals between successive flip-flops was then built and approximated by the sum of exponential functions:

(2) $$\begin{array}{c} P_{\text{flip}}\left(t\right)=A_{1}\exp\left(\frac{t}{t_{1}}\right)+A_{2}\exp\left(\frac{t}{t_{2}}\right), \end{array}$$

where *t*_1_ and *t*_2_ are the characteristic flip-flop times.

## 3. Results and Discussion

### 3.1. Membrane Shape and Curvature

**Figure 5:**
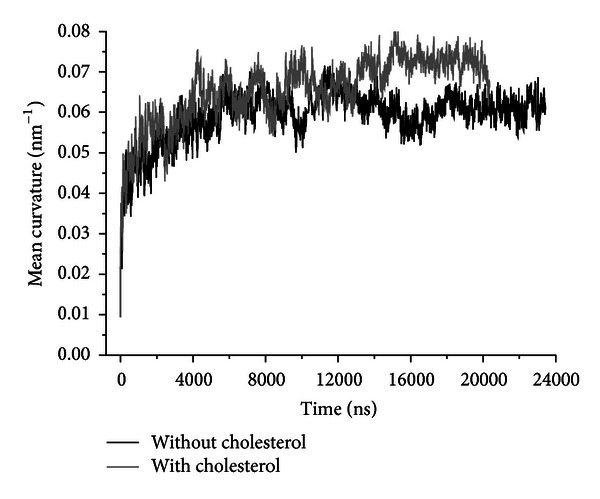
Evolution of the mean bilayer curvature in MD simulations.

During the simulation the bilayer developed significant curvature within each of asymmetric blocks and formed a highly bent “meander” structure (Figure 3(b)). Initial planar bilayer begins to bend immediately at the start of simulation. The mean curvature of the bilayer increases gradually (Figure 5) until the stable meander shape form is achieved after approximately 16 *μ*s. Equilibrium mean curvature of the bilayer without cholesterol is 0.06 ± 0.002 nm^−1^ (corresponding radius of curvature 16.7 nm). In the presence of cholesterol the mean curvature increases to 0.072 ± 0.002 nm^−1^ (radius of curvature 13.8 nm). 

### 3.2. Influence of Cholesterol on the Membrane Shape

**Figure 6:**
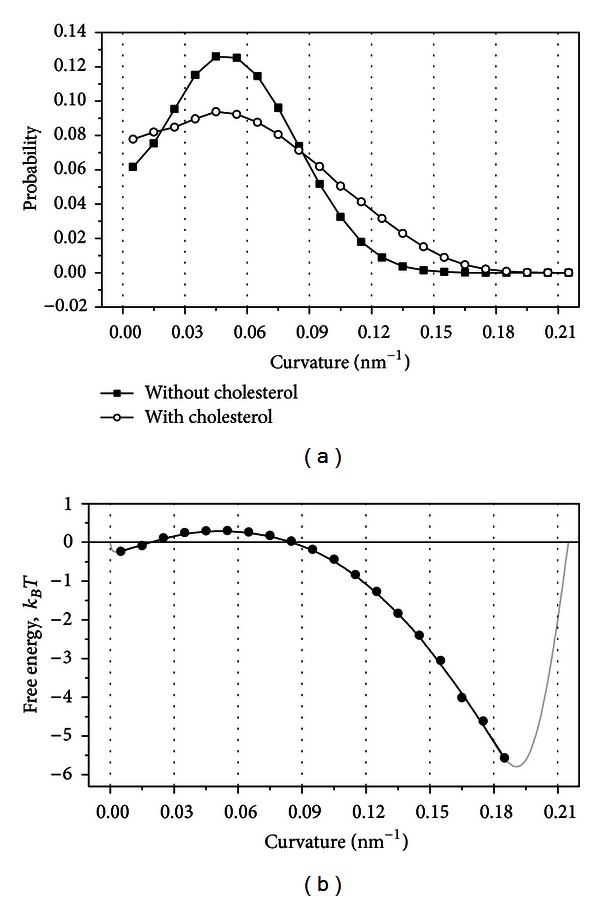
(a) Distributions of the membrane curvature in the simulation with and without cholesterol. (b) Free energy of transition from the equilibrium system without cholesterol to the equilibrium system with cholesterol as a function of the membrane curvature. Gray lines show hypothetical shape of the curve in the regions, which are not covered with data points. These lines are drawn for illustrative purposes in a qualitative manner and reflect the fact that the free energy profile must be limited by “walls” in order to insure the existence of steady states in the system.

In order to study in detail the influence of cholesterol on the membrane shape and curvature, we constructed the statistical distributions of the membrane curvature for the simulations with and without cholesterol (Figure 6(a)). In the case of the system without cholesterol the curvature was sampled at the discrete points of the membrane midline equally spaced by 1 nm on each trajectory frame. In the case of the system with cholesterol the curvature at the location of each cholesterol molecule was considered. It is clearly seen that the addition of cholesterol makes the distribution more uniform. The probability of finding the areas of very small (smaller than 0.017 nm^−1^) or very large (larger than 0.085 nm^−1^) curvatures is much higher in the presence of cholesterol. In contrast the probability of finding the average curvature around the peak of distribution (from 0.017 to 0.085 nm^−1^) decreases significantly in the presence of cholesterol.

 It is possible to compute the free energy of transition from the equilibrium system without cholesterol to the equilibrium system with cholesterol as a function of the membrane curvature *E*_Chol_ as

(3) $$\begin{array}{c} E_{\text{chol}}=-k_{B}T\ln\left(\frac{P_{\text{Chol}}\left(\kappa \right)}{P_{\text{no}\;\text{Chol}}\left(\kappa \right)}\right), \end{array}$$

where *T* = 320 K is the absolute temperature, *k*_*B*_ is the Boltzmann constant, and *P*_Chol_ and *P*_no Chol_ are the equilibrium distributions of the membrane curvature in the simulations with and without cholesterol, respectively. The plot of *E*_Chol_ is shown in Figure 6(b). Two energy wells on this potential correspond to the intervals of the membrane curvature, which become favorable upon the addition of cholesterol to the membrane. The energy well, which corresponds to very small membrane curvatures, is shallow and does not exceed 0.25*k*_*B*_ *T*. In contrast, the energy well corresponding to large curvatures reaches 5.5*k*_*B*_ *T*, which means that it would be populated significantly.

These results could be interpreted in the following way. In the absence of cholesterol the membrane is bent rather uniformly with the curvature close to the peak of distribution in Figure 6(a) (~0.05 nm^−1^). The most of the membrane has the radius of curvature around 20 nm while very sharp kinks (large curvature) and flat regions (small curvature) occupy only small parts of the membrane surface. In the presence of cholesterol the overall shape of the membrane changes dramatically. The amount of almost flat regions increases. These regions are separated by very sharp bends with very large curvature. The changes in the membrane curvature induced by cholesterol are shown schematically in Figure 8.

### 3.3. Influence of the Membrane Curvature on Cholesterol Distributions

The distribution of cholesterol molecules between the domains of DOPC and DOPS lipids *r*_DOPC/DOPS_ was measured for equilibrated part of MD trajectory using the procedure described in our previous work [7]. The value *r*_DOPC/DOPS_ = 0.65 was obtained. This confirms the result of our previous work [7] that in general cholesterol strongly prefers anionic lipids to neutral. However, it is interesting to see if this preference depends on the membrane curvature.

**Figure 7:**
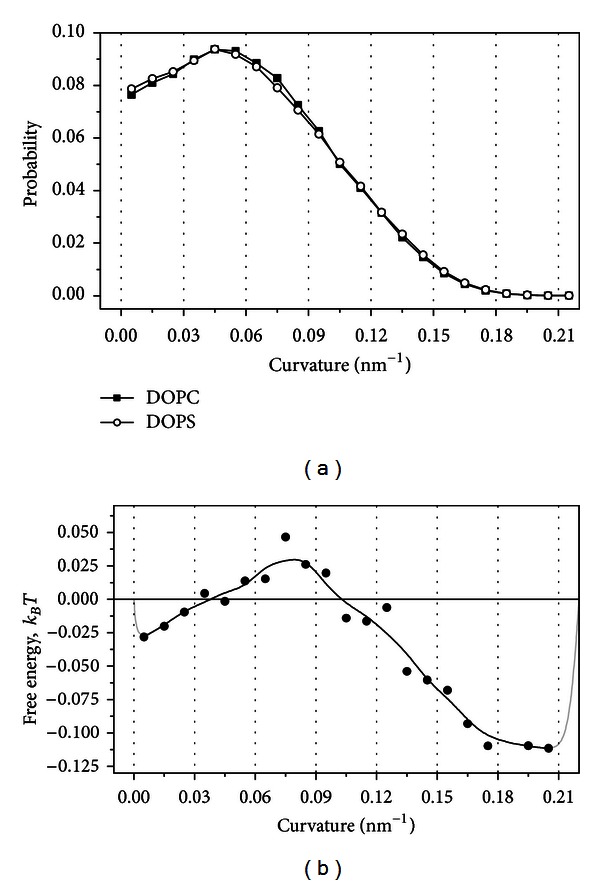
(a) Distributions of the membrane curvature in the individual lipid domains containing either DOPC or DOPS lipids measured in the locations of cholesterol molecules. (b) Free energy of transferring single cholesterol molecule from the lipid domain containing DOPC lipids to the domain containing DOPS lipids as a function of the membrane curvature. Gray lines show hypothetical shape of the curve in the regions, which are not covered with data points (see Figure 6 for explanation).

**Figure 8:**
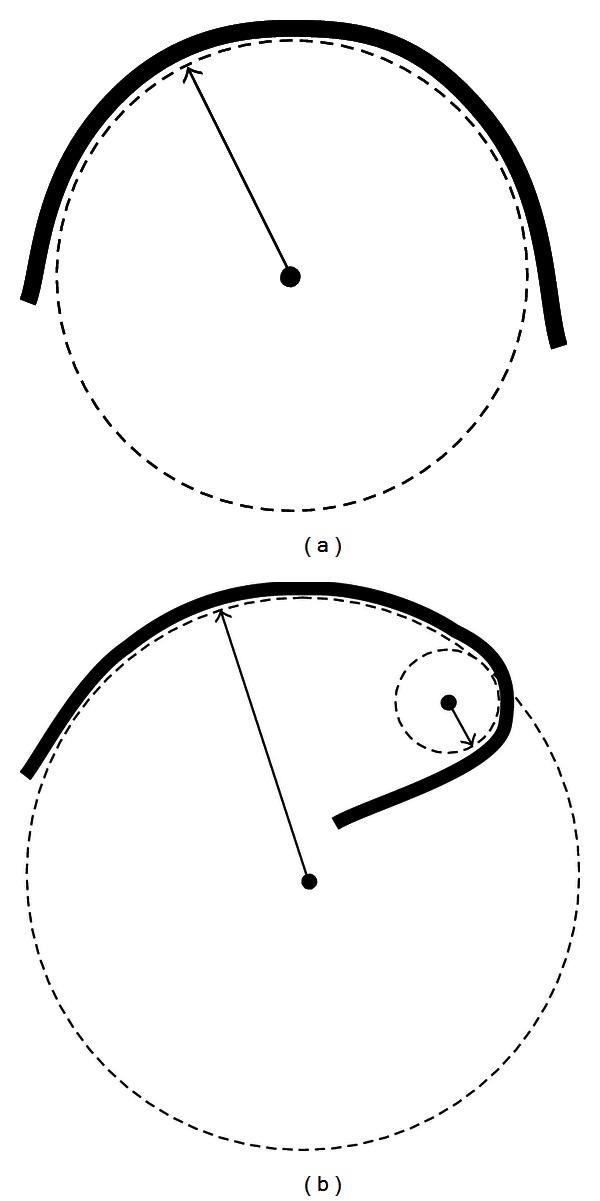
Schematic representation of evenly (a) and unevenly (b) curved bilayer. In (a) the whole bilayer patch is bent with almost the same radius of curvature. In (b) there are regions with very different radii of curvature. In our simulations the system without cholesterol is closer to the case (a) while the addition of cholesterol induces uneven curvature similar to (b).

We built statistical distributions of the membrane curvature measured in the locations of cholesterol molecules, which reside within individual lipid domains containing either DOPC or DOPS lipids (Figure 7(a)). The distributions for DOPC and DOPS lipids are very similar, but small differences exist. In order to visualize them the free energy of transferring single cholesterol molecule from the lipid domain containing DOPC lipids to the domain containing DOPS lipids could be computed as

(4) $$\begin{array}{c} E_{tr}=-k_{B}T\ln\left(\frac{P_{\text{DOPS}}\left(\kappa \right)}{P_{\text{DOPC}}\left(\kappa \right)}\right), \end{array}$$

where *P*_DOPC_ and *P*_DOPS_ are equilibrium distributions of the membrane curvature in the domains containing DOPC and DOPS lipids, respectively (Figure 7(b)). It is clearly seen that in the small range of curvatures from 0.04 to 0.1 nm^−1^ cholesterol molecules prefer DOPC lipid domains to DOPS domains (the transfer of cholesterol from DOPC to DOPS is energetically unfavorable). In contrast, for the curvatures smaller than 0.04 nm^−1^ or larger than 0.1 nm^−1^ cholesterol molecules prefer DOPS lipid domains to DOPC domains. These preferences are very small in terms of the free energy and do not exceed 0.1*k*_*B*_ *T*.

 Thus, the distribution of cholesterol molecules between the lipid domains containing DOPC or DOPS lipids is curvature dependent. In the almost planar or highly curved parts of the membrane the cholesterol prefers DOPS lipids, while in the moderately curved parts of the membrane it prefers DOPC lipids.

### 3.4. Flip-Flop Transitions of Cholesterol

 The histogram of the times between successive flip-flop transitions clearly suggests an existence of two very different flip-flop times (data not shown). Fitting this histogram with (2) reveals the characteristic flip-flop times *t*_1_ = 4.1 ± 0.0045 ns and *t*_2_ = 288.8 ± 0.34 ns. The time *t*_1_ is very close to the interval between successive frames in MD trajectory (4.0 ns), which is unlikely to be a coincidence. The most plausible explanation of this fact is the following. The flip-flop transition of cholesterol molecules is detected if the head group of cholesterol appears in one monolayer in the previous frame but in the other monolayer in the current frame. The orientation of cholesterol molecules and the depth where their head groups are located were not recorded for technical reasons. The cholesterol molecule located near the center of bilayer may “oscillate” between the monolayers multiple times until it leaves the hydrophobic core of the bilayer and exposes its head group into the water phase. Such very fast but spurious flip-flop transitions are likely to contribute to *t*_1_ and should be considered as an artifact. Thus only characteristic time *t*_2_ should be considered as true flip-flop time. The obtained flip-flop time of 288.8 ns is close to the lower limit of the flip-flop times estimated by Bennett et al. [13] in their study of symmetric coarse-grained DPPC bilayers (0.32–4.34 *μ*s) and of the same order of magnitude as obtained for DOPC/DOPS bilayers in our previous work [7]. 

## 4. Conclusion

The distribution of cholesterol in the highly curved asymmetric lipid bilayers containing DOPC and DOPS lipids was studied by the coarse-grained molecular dynamics simulations. A complex mutual influence of the membrane curvature, phospholipid distribution between the monolayers, and cholesterol distribution within and between these monolayers is revealed.

It was shown that cholesterol increases the average membrane curvature and induces uneven distribution of the membrane curvature. In the presence of cholesterol there appear a number of almost flat regions that are interleaved by the sharply bent areas. Such sharp bending is the feature introduced by cholesterol since in its absence the membrane curvature is rather smooth and uniform.

The role of cholesterol in modulating membrane curvature is particularly seen when its affinity to particular lipids is accounted. Although on average the cholesterol molecules prefer anionic DOPS to neutral DOPC lipids, this preference appears to be curvature dependent. The preference of cholesterol for DOPS lipids is observed either in almost planar or in highly curved parts of the membrane. In contrast, in the small interval of moderate curvatures the preference of the DOPC lipids is observed. It is necessary to note, however, that the corresponding free energies differences are small and thus may depend on the simulation parameters.

Our data suggest that there is a complex interplay between the membrane curvature, phospholipid distribution, and cholesterol distribution in the asymmetric lipid bilayers. This allows cholesterol to form uneven patterns of membrane curvature coupled to its uneven distribution in the plane of bilayer and between the monolayers.
